# Engineering *Komagataella phaffii* for secreted and surface-displayed production of SARS-CoV-2 receptor-binding domain and its immunogenicity evaluation

**DOI:** 10.1186/s12934-026-03043-5

**Published:** 2026-06-11

**Authors:** Kostyantyn Dmytruk, Olena Dmytruk, Marta Semkiv, Roksolana Vasylyshyn, Lyubov Fayura, Lidia Gaffke, Magdalena Podlacha, Zuzanna Cyske, Karolina Pierzynowska, Patrick Budylowski, Mario Ostrowski, Grzegorz Wegrzyn, Andriy Sibirny

**Affiliations:** 1 Department of Molecular Genetics and Biotechnology, Institute of Cell Biology, NAS of Ukraine, Drahomanov Street, 14/16, Lviv, 79005 Ukraine; 2 Department of Molecular Biology, Faculty of Biology, University of Gdansk, Wita Stwosza 59, Gdansk, 80-308 Poland; 3 Departments of Medicine, Immunology, University of Toronto, Medical Sciences Building, 1 King’s College Circle, Toronto, ON M5S 1A8 Canada; 4 Faculty of Biotechnology, Medical College, University of Rzeszów, Cwiklinskiej 2D, Rzeszów, 35-601 Poland

**Keywords:** Yeast expression system, Antigen surface display, Neutralizing antibody response, Oral vaccine delivery

## Abstract

**Background:**

The receptor-binding domain (RBD) of the SARS-CoV-2 spike protein represents a key antigen for vaccine development due to its critical role in the ACE2 receptor recognition. Yeast-based expression systems, particularly *Komagataella phaffii*, offer scalable and cost-effective platforms for recombinant protein production. In addition to secretion, cell surface display provides an alternative strategy enabling direct antigen delivery, including applications via mucosal routes.

**Results:**

In this study, we engineered *K. phaffii* strains for the production of SARS-CoV-2 RBD in two formats: as a secreted recombinant protein and as a cell surface-displayed antigen using a Sag1 anchoring system. Incorporation of glycine–serine linkers enhanced secretion efficiency, yielding up to 50 mg/L of RBD, with minimal intracellular retention. Western blot analysis indicated the presence of glycosylated forms of RBD, and the recombinant protein was subsequently purified for further characterization. Surface localization of RBD was validated by immunofluorescence microscopy and quantitative fluorescence measurements. Immunogenicity studies in mice demonstrated that intraperitoneal administration of purified RBD elicited a strong humoral immune response. Importantly, sera from immunized animals efficiently inhibited spike protein binding to the Ace2 receptor, indicating potent neutralizing activity. Comparable results were obtained following oral administration of *K. phaffii* cells displaying RBD on their surface, demonstrating the feasibility of a whole-cell yeast-based vaccine approach.

**Conclusions:**

A yeast-based system enabling both secretion and surface display of SARS-CoV-2 RBD has been developed. Both delivery strategies, intraperitoneal administration of the purified protein and oral administration of recombinant yeast cells, induced robust and functionally relevant immune responses in mice. These findings highlight the potential of *K. phaffii* as a versatile system for the development of cost-effective subunit and oral vaccine candidates.

**Supplementary Information:**

The online version contains supplementary material available at 10.1186/s12934-026-03043-5.

## Introduction

Severe Acute Respiratory Syndrome Coronavirus 2 (SARS-CoV-2) triggered a major global epidemic from 2020 [1] until 2023 [2], resulting in approximately 7 million deaths worldwide, while over 760 million individuals were infected, according to World Health Organization data [3]. Current evidence suggests that SARS-CoV-2 is transitioning toward endemicity, exhibiting seasonal patterns similar to influenza [4]. Vaccination has remained and continues to be a highly effective strategy to prevent SARS-CoV-2 infection and mitigate severe disease outcomes [5]. During the pandemic, a variety of vaccine platforms were developed and deployed, with mRNA vaccines, adenovirus-based vaccines, inactivated virus vaccines, and protein subunit vaccines occupying leading roles [6]. Among these approaches, the development of oral vaccines based on microorganisms with surface-displayed antigens has emerged as a promising strategy.

Microbial cells displaying viral antigens on their surface represent a promising platform for vaccine development. In this context, genome-reduced *Escherichia coli* expressing a recombinant gene coding for the SARS-CoV-2 fusion peptide exposed on the cell surface, as well as attenuated *Francisella tularensis* producing the coronavirus M and N proteins have been proposed as vaccine candidates, demonstrating protective efficacy in porcine and hamster models, respectively [7, 8]. Similarly, *Lactiplantibacillus plantarum* engineered to display SARS-CoV-2 epitopes has been explored as a mucosal vaccine, eliciting both humoral and mucosal immune responses in mice [9]. Recombinant yeast strains have also been employed. Namely, *Saccharomyces cerevisiae* displaying the receptor-binding domain (RBD) of the Spike protein [10] and *Komagataella phaffii* producing a recombinant antigen composed of Spike and Nucleocapsid epitopes [11] have successfully induced immune responses following oral administration.

To enhance the stability and activity of surface-displayed antigens under diverse environmental conditions, spore-based vaccines have been developed. Specifically, *Bacillus subtilis* spores displaying the RBD domain of SARS-CoV-2 have been proposed as an oral vaccine prototype [12], while *S. cerevisiae* spores presenting RBD have shown high gastrointestinal stability in vitro and elicited effective humoral and mucosal immunity in mice [13]. Collectively, those studies highlighted the versatility and potential of microbial surface display systems for the development of safe, orally deliverable vaccines.

As indicated above, the feasibility of microbial cell–based vaccines employing surface display of SARS-CoV-2 antigens across diverse biological platforms, including bacteria, bacterial spores, and yeasts was demonstrated. While bacterial systems enable rapid antigen presentation and can elicit protective immune responses, yeast-based platforms offer several distinct advantages that are particularly relevant for oral vaccination strategies [14]. Yeasts exhibit high intrinsic resistance to the harsh conditions of the gastrointestinal tract, including fluctuations in pH and exposure to digestive enzymes, thereby ensuring improved antigen stability and delivery to mucosal immune sites [15]. In addition, yeast cell walls possess natural immunostimulatory properties, which may reduce or eliminate the need for external chemical adjuvants, lowering both production complexity and cost [16]. Furthermore, yeasts have been extensively exploited as biofactories for heterologous protein production, with *S. cerevisiae* and *K. phaffii* being the most widely used species, both of which hold GRAS (generally recognized as safe) status [17, 18]. *Komagataella phaffii* is particularly distinguished by its high recombinant gene expression capacity, robust growth on simple media, and ability to perform post-translational modifications resembling those of higher eukaryotes [19]. Taken together, these properties make yeast-based surface display systems highly attractive and scalable platforms for the development of vaccines. Importantly, oral vaccines leveraging these systems combine a favorable safety profile, low production cost, and ease of distribution, with generally higher acceptance and tolerability by recipients, compared to injectable formulations, reflecting a preference for non-invasive routes of administration [20–23].

Following oral administration, yeast cells displaying heterologous antigens interact with the host immune system primarily through innate immune recognition pathways. Yeast-exposed pathogen-associated molecular patterns (PAMPs), including β-glucans, mannans, and chitin, are recognized by pattern recognition receptors such as Toll-like receptors and C-type lectin receptors, including Dectin-1, located on intestinal dendritic cells and macrophages [21, 24]. These interactions promote phagocytosis of yeast cells and subsequent antigen processing. Antigen presentation within gut-associated lymphoid tissues, particularly Peyer’s patches and mesenteric lymph nodes, supports the activation of CD4⁺ T cells and drives B-cell class switching toward secretory IgA production, resulting in mucosal immune protection. In parallel, activated antigen-presenting cells migrate to secondary lymphoid organs, where antigen presentation contributes to systemic immune priming. This process supports the generation of antigen-specific IgG antibodies and Th1-type CD4⁺ T-cell responses through MHC class II pathways. Together, the coordinated induction of mucosal IgA and systemic IgG responses, along with Th1 polarization, underlies the effectiveness of yeast-based oral vaccines as self-adjuvanting platforms capable of eliciting both local and systemic immunity [22].

In this study, we engineered *K. phaffii* cells to display the SARS-CoV-2 spike protein receptor-binding domain (RBD) on their surface. The novelty of our approach lies in the use of a humanized *K. phaffii* strain, combined with a direct comparative evaluation of immune responses elicited by intraperitoneal administration of the purified, secreted RBD protein versus oral delivery of yeast surface-displayed RBD in mice. This dual strategy provides a comprehensive assessment of the potential of surface-immobilized RBD in eliciting systemic and mucosal immunity, highlighting the advantages of yeast-based platforms for vaccine development.

## Materials and methods

### Plasmids and microbial strains

**Fig. 1:**
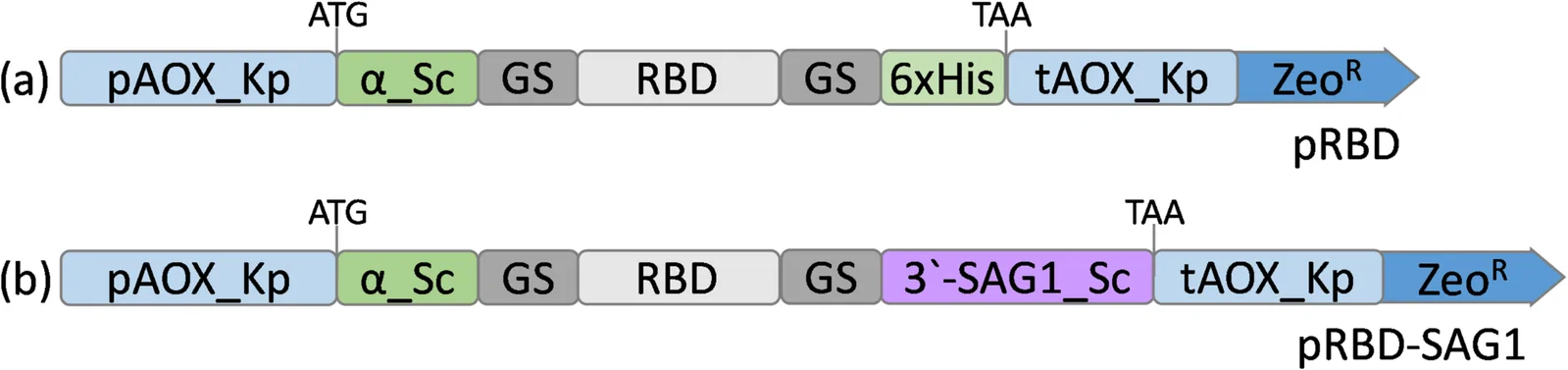
Linear scheme of plasmid **a** pRBD, and **b** pRBD-SAG1. Promoter and terminator of the *AOX K. phaffii* are indicated as blue boxes; RBD - as grey box; glycine- and serine-rich linkers – as dark grey boxes; *S. cerevisiae* α-factor secretion signal and 6×His tag – as green boxes; *S. cerevisiae* 3`-*SAG1* gene – violet box; selective marker Zeo^R^– as dark blue boxes, respectively

For expression of a gene coding for an RBD fragment in *K. phaffii*, the pPICZαB expression vector (Thermo Scientific) was used as the base plasmid. This vector carries the methanol-inducible alcohol oxidase (AOX) promoter from *K. phaffii* and the *S. cerevisiae* α-factor secretion signal, enabling efficient secretion of the recombinant RBD into the culture medium. The RBD (from 331 to 529 amino acids) of surface glycoprotein of SARS-CoV-2 protein sequence (GenBank accession number QIA98554.1; https://www.ncbi.nlm.nih.gov/protein/QIA98554.1) was used as the reference sequence. Codon optimization for *K. phaffii* was performed using the ATGme online tool (http://atgme.org/) to match the codon usage preferences of the yeast genome. To enhance secretion efficiency, the RBD-coding sequence was flanked by flexible glycine- and serine-rich (GS)-encoding linker sequences. In addition, in the recombinant gene product, a 6×His affinity tag, separated by a TEV protease cleavage site, was fused to the C-terminus of the RBD to facilitate purification by metal affinity chromatography. The resulting plasmid containing the synthetic construct, designated pRBD, was generated by BioCat (https://www.biocat.com/) (Fig. 1a). The complete sequence of pRBD is shown in Fig. S1.

For cell-surface display, the RBD-coding sequence was fused to a DNA sequence encoding the GPI-anchor domain, derived from the 3’-terminus of the *S. cerevisiae SAG1* gene, encoding α-agglutinin. A DNA fragment coding for GS-flanked RBD was amplified from pRBD using primers Ko1160 and Ko1161 (sequences of all used primers are shown in Table S1). The *SAG1* anchor fragment was amplified from genomic DNA of *S. cerevisiae* S288C using primers Ko1152 and Ko1153. Both fragments were digested with EcoRI/XbaI (*RBD*) and XbaI/SalI (*SAG1*), and ligated into EcoRI/SalI-digested pPICZαB. The resulting plasmid was designated pRBD-SAG1 (Fig. 1b) and verified by sequencing. The nucleotide sequence of pRBD-SAG1 is shown in Fig. S2.

*E. coli* DH5α [Φ80d*lac*ZΔM15, *recA*1, *endA*1, *gyrA*96, *thi-*1, *hsdR*17(r^−^_K_ m^+^_K_), *supE*44, *relA*1, *deoR*, Δ(*lacZYA-argF*) U169] was used for routine plasmid cloning and propagation. Cultures were grown at 37 °C in LB medium as previously described [25]. Transformed *E. coli* cells were maintained in rich medium containing 50 mg/L of zeocin.

Plasmids pRBD and pRBD-SAG1 were linearized using SacI and BglII, respectively and introduced by transformation into the *K. phaffii* GlycoSwitch SuperMan5 strain (https://pichia.com/try-pichia/try-pichia-glycoswitch/). Transformation of *K. phaffii* was performed by electroporation according to a standard protocol, described previously [26]. Transformants were selected on YPD agar plates supplemented with 500 µg/mL zeocin.

Transformant stabilization was achieved by alternating 15–20 generations of cultivation in non-selective and selective media. The resulting stable strains KpRBD and KpRBD-SAG1 were verified by colony PCR using pairs of primer Ko1104/Ko1155 and Ko1162/Ko1163 to confirm the presence of the *RBD* and *RBD-SAG1* genes.

### Molecular-biology techniques

Standard cloning techniques were used as described by ref. [25]. Genomic DNA of yeasts was isolated using the NucleoSpin^®^ Tissue Kit (Macherey-Nagel). Restriction endonucleases and DNA ligase (Thermo Scientific) were used. Plasmid isolation from *E. coli* was performed by GeneJET Plasmid Miniprep Kit (Thermo Scientific). The target fragments were PCR-amplified using Phusion High-Fidelity DNA Polymerase (Thermo Scientific) in a Mastercycler nexus X2 thermal cycler (Eppendorf).

### Cultivation condition of engineered *K. phaffii*

To induce RBD secretion, yeast strain KpRBD was pre-cultured from fresh agar plates in YPG medium (0.5% yeast extract, 1% peptone, 1% glycerol) for 24 h. Cultures were then inoculated at a starting OD_600_ of 5.0 into 100 mL of YPM medium (0.5% yeast extract, 1% peptone, 1% methanol) in 300 mL Erlenmeyer flasks and incubated at 30 °C for 120 h with shaking at 200 rpm. Methanol was supplemented every 24 h to a final concentration of 1%. The culture medium was separated from cells by centrifugation, supplemented with 1 mM PMSF, and stored at − 80 °C for subsequent RBD purification. To induce cell surface immobilization of RBD, the KpRBD-SAG1 strain and the control strain (Control) carrying the plasmid vector pPICZαB were cultivated in YPM medium supplemented with 1% methanol for 72 h at 30 °C, starting from an initial OD₆₀₀ of 0.3. Cells were harvested and washed three times with sterile PBS. Aliquots (100 µL) of cell suspensions adjusted to an OD₆₀₀ of 100 were heat-inactivated at 60 °C for 10 min. Complete inactivation was confirmed by plating 30 µL of the treated suspensions onto YPD agar plates and verifying the absence of colony formation. The inactivated cells were stored at − 80 °C until further analysis. Yeast biomass was quantified turbidimetrically by measuring optical density at 600 nm using a Helios Gamma spectrophotometer (10-mm path-length cuvette) and calibrated against gravimetric measurements. Cell concentration was estimated using a conversion factor of 1 OD₆₀₀ corresponding to 5 × 10⁷ cells/mL, according to the *Pichia pastoris* expression kit manual.

### Biochemical methods

Total cell extracts were prepared for immunoblotting as described previously [27]. Proteins secreted into the culture medium were collected after 120 h of cultivation and concentrated 50-fold using a Vivaspin 20 centrifugal concentrator equipped with a 10 kDa molecular weight cut-off PES membrane. For analysis, 50 µL of the concentrated medium was mixed with an equal volume of 2×SDS sample buffer and boiled for 5 min prior to electrophoresis. SDS–polyacrylamide gel electrophoresis (SDS–PAGE) was performed as described previously [28], followed by Western blotting according to established protocols [29]. Protein detection was carried out using an enhanced chemiluminescence reagent (LumiGO ECL Substrate Kit; Cleaver Scientific) after incubation with an anti–SARS-CoV-2 Spike RBD antibody (Sigma).

### Protein purification

Protein purification was performed by immobilized metal affinity chromatography (IMAC). The concentrated culture medium was incubated with NEBExpress™ Ni Resin (New England Biolabs) at a ratio of 1 mL of medium to 300 µL of a 50% resin suspension, with binding carried out at 4 °C for 18 h under gentle agitation. Purification was then conducted using NEBExpress™ Ni Spin Columns according to the manufacturer’s instructions. Bound proteins were eluted with 300 mM imidazole. The eluate was dialyzed for 18 h against 50 mM potassium phosphate buffer (pH 7.5) at 4 °C with gentle stirring, using an appropriate dialysis membrane. The purified protein was sterilized by filtration through a 0.2 μm Minisart^®^ RC 4 membrane filter (Sartorius) and stored at − 80 °C for subsequent Western blotting analysis and in vivo immunization studies. Protein concentrations were determined spectrophotometrically by measuring absorbance at 280 nm, using the corresponding molar extinction coefficients.

### Immunofluorescence assay

For immunofluorescence labeling, 200 µL of cell suspensions adjusted to an OD₆₀₀ of 1.0 were incubated with primary antibodies against the SARS-CoV-2 Spike RBD (Sigma) at a 1:400 dilution for 3 h. The cells were subsequently washed three times with PBS and incubated for 2 h with fluorescently labeled secondary antibodies (anti-rabbit IgG (H + L), CF488A; Sigma-Aldrich) at a 1:400 dilution. Following incubation, cells were washed three additional times with PBS. Fluorescence imaging was performed using an Axio Imager A1 microscope (Carl Zeiss MicroImaging) equipped with a monochrome digital camera (AxioCam MRm). Images were processed using AxioVision 4.5 software and ImageJ (version 1.53). For quantitative assessment of RBD immobilization on the cell surface, fluorescence intensity was measured using an RF-6000 spectrofluorometer (Shimadzu) at excitation and emission wavelengths of 490 nm and 515 nm, respectively. Measurements were conducted in aqueous suspensions of labeled cells adjusted to an OD₆₀₀ of 0.2.

### Animals

Male CBA/J mice (Tri-City Central Animal Laboratory, Research Service Center of the Medical University of Gdansk, Poland) were used in in vivo experiments. Animals were kept in a ventilated animal facility, with constant artificial lighting cycle (12 h light/12 h dark), ambient temperature (22 °C), and constant air humidity (55%). Access to food and tap water was *ad libitum*.

All experiments were carried out with consent of the Local Ethical Committee for the Care and Use of Laboratory Animals in Bydgoszcz, Poland (resolution number 17/2018). The animal house met the requirements of the Law on the Protection of Animals Used for Scientific or Educational Purposes (Journal of Laws, dated on February 26, 2015), as well as the recommendations of the European Commission on the welfare of animals used in scientific experiments. Mice were housed in polycarbonate cages (20 cm wide, 37 cm long, and 18 cm high) and were allowed to adapt to the experimental conditions for 1 week.

Mice were 6 months old at the start of the experiments and weighed between 34 and 37 g. Each group of mice receiving a particular preparation by intraperitoneal administration consisted of 8 individuals, while those receiving oral administration consisted of 12 animals per group.

### Immunization

For intraperitoneal administration, 50 µg protein in 100 µl buffered saline (PBS) or 100 µg of yeast cells in 100 µl PBS per mouse were used for immunization. For oral administration performed with a metal gavage, 100 µg of yeast cells in 150 µl PBS per mouse was used. The dose of yeast cells used for oral immunization was selected based on amounts reported in previous studies employing *S. cerevisiae* with surface-displayed RBD as an antigen delivery platform [10]; however, the amount of surface-displayed RBD associated with this biomass was not quantified, and therefore the exact antigen dose delivered to the animals remains unknown. A booster dose was given 4 weeks after the first administration.

### Blood collection

Blood was collected from the venous plexus inside the orbit behind the eyeball, using a sterile disposable capillary (2 cm long and 1 mm diameter) into tubes with a gel separator and coagulation activator. For analysis, 400–500 µl of blood was collected, depending on the weight of the animal. Throughout the procedure, the animals were anesthetized with a mixture of ketamine (87.5 mg/kg body weight) and xylasine (12.5 mg/kg body weight). The collected blood was centrifuged (1800–2200 x g, 10 min) to obtain serum. Then, the sera were inactivated at 56 °C for 30 min.

### Determination of levels of neutralizing antibodies

A neutralization assay was used to determine the level of antibodies. The procedure was carried out using a commercial kit (Life Technologies, #BMS2326) according to manufacturer’s instruction. All reagents and samples were brought to room temperature (20–25 °C) before use. Each well of the titration plate (96-well Nunc plate), was washed twice with the previously prepared buffer (included in the kit). Then, 100 µl of (i) positive control, (ii) assay buffer (a negative control), or (iii) test sample diluted 1:50, were added. The plate was covered and incubated at room temperature for 30 min with shaking. Next, the plate contents were drained and washed with the prepared buffer three times, followed by addition of 100 µl of biotin conjugate solution. The plate was covered and incubated again at room temperature with shaking, and plate contents were drained and washed with prepared buffer three times. Subsequently, 100 µl of streptavidin-labeled horseradish peroxidase enzyme solution was added and the plate was incubated for 15 min at room temperature with shaking. The reaction was stopped by adding 100 µl of the blocking solution, which changed the color of the product (from blue to yellow). Absorbance was measured 5 min after stopping the reaction, using an Enspire microplate reader (Enspire Multimode Plate Reader, PerkinElmer, USA).

### Statistical analysis

Quantitative results are presented as mean ± SD (standard deviation). For statistical analysis of the results, SPSS 21.0 (SPSS Inc., Amonk, USA) software was used. The normality of the distribution of variables was checked with the Kolmogorov-Smirnov test, and the homogeneity of the variances with the Levene test. Since the assumptions authorizing parametric analysis were not met, the Kruskal-Wallis and Dunn tests were used. The *p* value lower than 0.05 was considered statistically significant.

## Results and discussion

### Design of RBD expression vectors and generation of recombinant yeast strains

To enable the expression of *RBD* in *K. phaffii*, we designed codon-optimized nucleotide sequence and constructed vectors for both secreted and surface-displayed forms of the protein. Expression in *K. phaffii* was driven by the methanol-inducible AOX promoter, and vectors included DNA sequences coding for an *S. cerevisiae* α-factor secretion signal at the N-terminus, along with a 6 × His tag for purification. To enhance secretion efficiency, flexible glycine–serine-encoding linkers were incorporated at both ends of the RBD-coding sequence, yielding GS-tagged constructs (Fig. 1a). For surface immobilization, the RBD was fused in-frame with the C-terminal domain of the *S. cerevisiae* cell wall protein α-agglutinin (Sag1), enabling GPI-anchor-mediated localization (Fig. 1b). All plasmids were sequence-verified and introduced by transformation into glycoengineered yeast strain *K. phaffii* SuperMan5 with humanized N-glycosylation profiles. Stable integrants were selected and verified via colony PCR. Between 10 and 20 colonies of each type of transformants were analyzed to identify those with the highest levels of target protein secretion or surface immobilization. The selected strains were designated as KpRBD for protein secretion, and KpRBD-SAG1 for surface display of RBD. These strains were used for subsequent studies.

### RBD secretion and purification

**Fig. 2:**
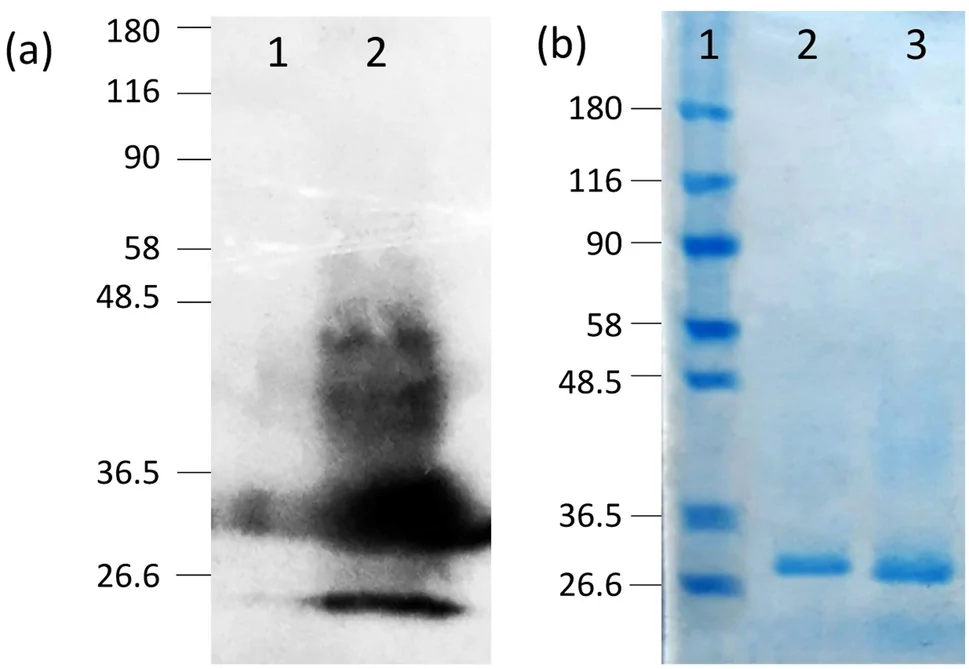
Western-blot and SDS-PAGE analysis of RBD expression in *K. phaffii*. Western-blot analysis of cell extracts (1) and culture supernatants (2) of KpRBD *K. phaffii.* Anti-RBD polyclonal antibody (Sigma) was used for Western-blot (**a**); the molecular weight marker is shown on the left in kDa (1). SDS–PAGE of purified recombinant RBD with 0.2 (2) and 0.5 µg (3) of protein loaded (**b**)

*K. phaffii* strains were cultivated under methanol induction in YPM medium, and RBD production was evaluated in both culture supernatants and cell extracts. The incorporation of glycine–serine linkers resulted in efficient RBD secretion (50 mg/L), with only a minor fraction of the recombinant protein retained intracellularly (Fig. 2a). During Western blotting analysis of the culture medium, several bands were detected. The theoretically calculated molecular mass of the GS-tagged RBD variant is approximately 26.6 kDa. The RBD contains two N-linked glycosylation sites (N331 and N343), which are known to result in heterogeneous glycosylation when present in yeast [30]. The dominant band at ~ 30 kDa corresponds to a mono-glycosylated form of RBD (Fig. 2a), whereas additional bands likely reflect glycosylation at both sites.

The more homogeneous, humanized N-glycosylation profile of the glycoengineered *K. phaffii* SuperMan5 strain may contribute to improved protein stability and solubility, consistent with previous reports demonstrating the impact of glycosylation on protein folding, stability, and physicochemical properties [18, 31]. This highlights the potential of glycoengineered yeasts for producing therapeutically relevant RBD while indicating areas for further optimization of glycosylation profiles.

The recombinant protein excreted into the culture YPM medium was purified using nickel-affinity chromatography under native conditions via its C-terminal His-tag. Eluted fractions of the secreted RBD were analyzed by SDS–PAGE (Fig. 2b). The molecular weight of the purified protein corresponded to the dominant band observed in the Western blotting analysis (Fig. 2).

### Surface display of RBD

**Fig. 3:**
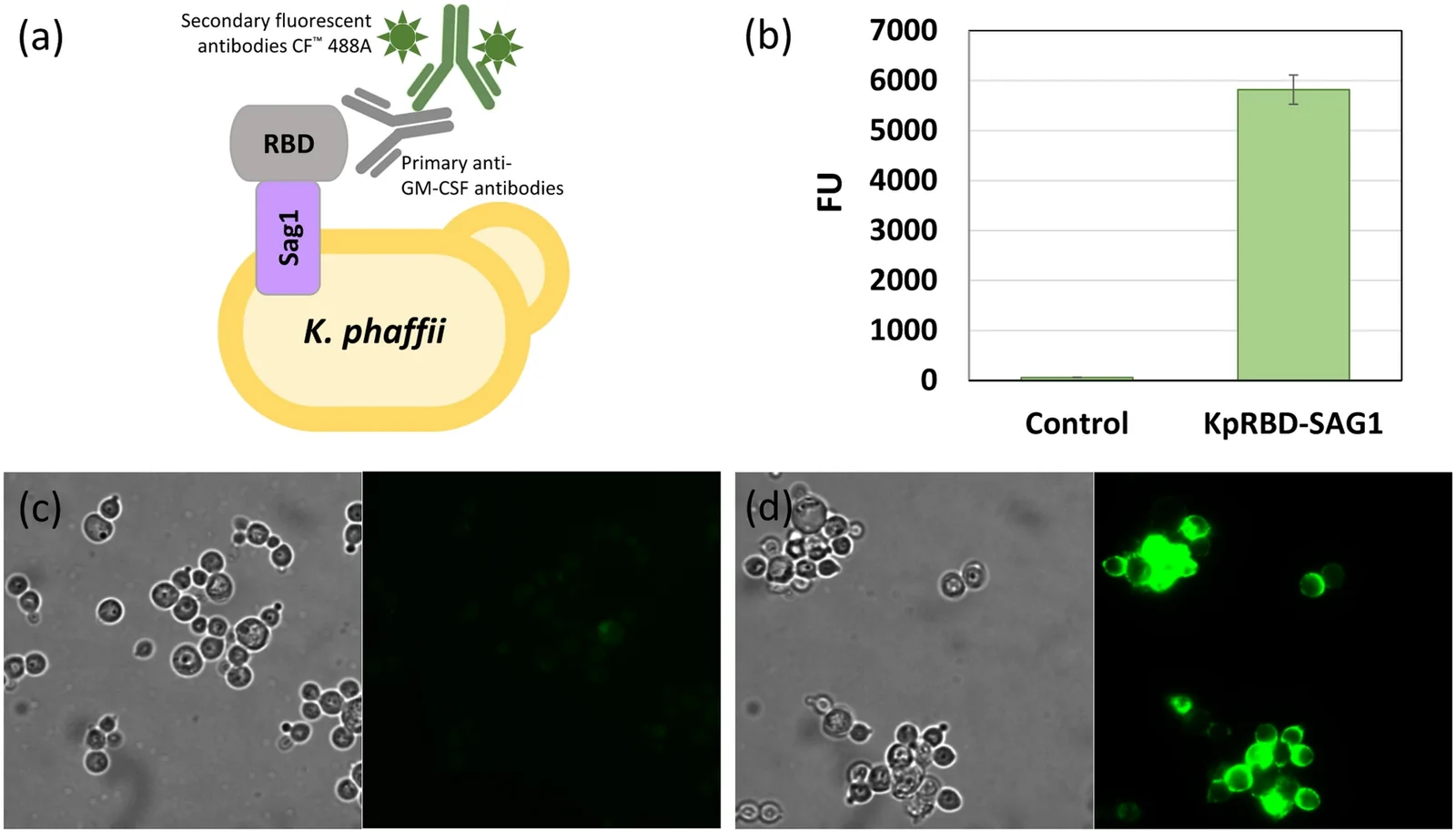
Surface display of RBD in yeast: schematic, fluorescence quantification, and microscopy. Schematic representation of RBD surface display in yeast (**a**); fluorescence of control strain SuperMan5 carrying empty vector and KpRBD-SAG1 grown on methanol-supplemented rich (YPM) medium and labeled with primary anti-RBD antibodies and CF™ 488 A-conjugated secondary antibodies using an RF-6000 spectrofluorometer (Shimadzu) with excitation and emission wavelengths of 490 nm and 515 nm, respectively (**b**); fluorescence microscopy images of control strain (**c**) and KpRBD-SAG1 (**d**)

Immunofluorescence analysis was performed for the *K. phaffii* strain KpRBD-SAG1 producing the RBD–SAG1 fusion protein. The principle of the assay is illustrated in Fig. 3a, showing the use of primary anti-RBD antibody, followed by CF™ 488 A-conjugated secondary antibody. The fluorescence intensity of this strain exhibited almost 100-fold increase in fluorescence, compared to the control strain bearing empty vector (Fig. 3b), i.e. 5820 FU versus 60 FU. In immunofluorescence analysis, the control strain displayed fluorescence levels comparable to autofluorescence (cells without antibody treatment or with secondary antibody only), confirming the specificity of antibody binding to the target epitope. Surface localization of RBD was confirmed by indirect immunofluorescence microscopy, with a detectable fluorescence signal in approximately 90% of the cells, indicating successful display via the GPI-anchor (Fig. 3c, d). This surface display approach offers a purification-free alternative for RBD delivery and may serve as a platform for direct interaction with target immune cells.

### Neutralizing activity of induced antibodies

To determine neutralizing activities of purified RBD and cells surface-displaying the RBD, a commercially available neutralization assay was used (described in detail in Materials and Methods section). It is usually used to assess the immune response after SARS-CoV-2 infection or vaccination against COVID-19. If neutralizing antibodies are present in the test sample, they compete with the biotinylated ACE2 receptor for binding sites on the solid-phase-coated S1 antigen of SARS-CoV-2. Unbound ACE2 is removed at the wash step, preventing the binding of the conjugate horseradish peroxidase-labeled streptavidin to the biotinylated ACE2 receptor, and the occurrence of a color reaction after substrate addition. In the absence of neutralizing antibodies in the test sample, the biotinylated ACE2 receptor binds to the S1 antigen of SARS-CoV-2, allowing the reaction to proceed further, i.e. to bind particular reagents and form a reaction complex. The result is a color reaction indicating the binding of ACE2 to the S1 antigen.

**Fig. 4:**
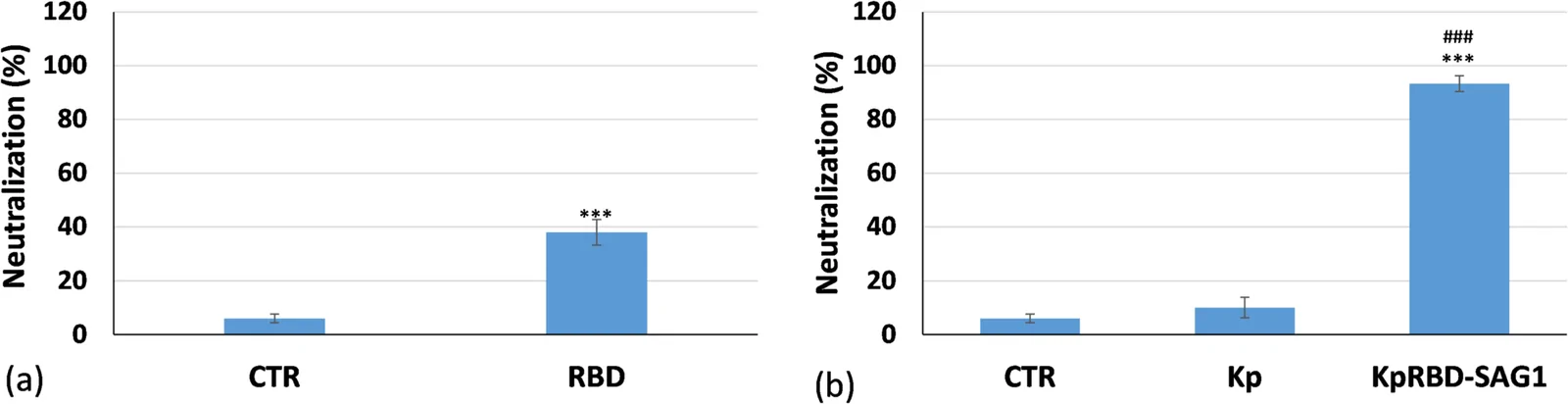
Neutralizing antibody responses to purified and yeast-displayed RBD in mice. Levels of anti-SARS-CoV-2 neutralizing antibodies after immunization with the purified RBD preparations administered intraperitoneally, relative to the non-immunized control group (CTR) (**a**). Levels of anti-SARS-CoV-2 neutralizing antibodies following oral immunization with KpRBD-SAG1 cells surface-displaying the RBD, relative to the non-immunized control group (CTR) or the control strain bearing empty vector (Kp) (**b**). Statistically significant differences between tested preparations and the control group are indicated as follows: *** *p* < 0.001, vs. CTR, ### *p* < 0.001 vs. Kp

Intraperitoneal administration of purified RBD to mice resulted in 38% levels of anti-SARS-CoV-2 neutralizing antibodies compared with the control group (*p* ≤ 0.001; Fig. 4a). Statistical analysis using the Kruskal–Wallis test (H(2,15) = 91.011, *p* ≤ 0.001) and Dunn test confirmed the significance of these differences.

In the case of oral administration, the levels of neutralizing antibodies after immunization with cells surface-displaying the RBD were analyzed. The Kruskal-Wallis test (H2,10 = 61.198, *p* ≤ 0.001) and Dunn test revealed a statistically significant increase in neutralizing antibody levels, reaching 93% after administration of KpRBD-SAG1 (*p* ≤ 0.001), compared with both the non-immunized control group and the control strain carrying an empty plasmid (Fig. 4b).

The markedly higher neutralizing antibody response observed following oral administration of KpRBD-SAG1-displaying cells (93% of control levels) compared to intraperitoneal administration of purified RBD (38%) may reflect, at least in part, the adjuvant properties of whole yeast cells. Yeast cell wall components, such as β-glucans and mannans, are known to activate pattern recognition receptors (e.g., TLR2/4 and Dectin-1) on antigen-presenting cells, thereby enhancing both innate and adaptive immune responses [32]. However, as the amount of surface-displayed RBD was not quantified, differences in antigen dose between the two delivery formats cannot be excluded as a contributing factor to the observed effect. It should be noted that the presented here results are reproducible using only described biochemical and immunological reactions as well as the murine lines used.

A limitation of the present study is the lack of quantification of surface-displayed antigen and the absence of data on its stability during gastrointestinal transit. These parameters are important for precise dose definition and for evaluating the efficiency of oral antigen delivery. Future work should address (i) quantitative assessment of displayed antigen levels, (ii) evaluation of antigen stability under simulated gastric conditions, and (iii) dose–response analyses to optimize immunization regimens.

## Conclusions

High level production system of SARS-CoV-2 RBD was developed in humanized *K. phaffii*, allowing efficient secretion or surface display of the recombinant proteins. Administration of both the RBD preparations and yeast cells exposing this protein on their surfaces to mice, either intraperitoneally or orally, resulted in appearance of neutralizing antibodies with high efficiency in selected preparations. Therefore, the developed *K. phaffii* system allows for production of cost-effective subunit and oral vaccine candidates.

## Electronic Supplementary Material

Below is the link to the electronic supplementary material.

Supplementary Material 1.

## Data Availability

All data generated or analyzed during this study are included in this published article and its supplementary information files. Additional data are available from the corresponding author on reasonable request.
