# Supplementary material for: Engineering Komagataella phaffii for secreted and surface-displayed production of SARS-CoV-2 receptor-binding domain and its immunogenicity evaluation
Source: Microb Cell Fact. 2026 Jun 11;25:174. doi: 10.1186/s12934-026-03043-5 (PMC13491813; doi:10.1186/s12934-026-03043-5)
Supplement: Supplementary file 1 [file 12934_2026_3043_MOESM1_ESM.docx]

**Table S1 List of primers used in this study (restriction sites are underlined)**

| **Primer name** | **Primer sequence 5’-3’** |
| --- | --- |
|  |  |
| Ko1104 | CTTTCATAATTGCGACTGGTTC |
| Ko1152 | ACT TTT CTA GAG GTC GGA ACC TCG GTA CAG |
| Ko1153 | GCT ACA AAC GTC GAC TTA GAA TAG CAG GTA CGA CAA AAG C |
| Ko1155 | CCG ACC TCT AGA AAA GTT AAC ACA CTT GTT CTT AAC CAA G |
| Ko1160 | CCG GAA TTC TTG GTG GTT CTT CTG GTG GTT CTA ACA TTA CTA ACT TGT GTC CAT TTG G |
| Ko1161 | GCT CTA GAG CGG CCG CCG GTA CCA GAA GAA GAA GAA GAA GAA GAA GAA GAA GAA CCA CCA GAA CCA CCC TTC TTT GGA CCA CAA ACA GTA G |
| Ko1162 | CTT GTA CAG ATT GTT TAG AAA GTC |
| Ko1163 | CTG TTT CTC TGC TAA TGG TTT C |
|  |  |

Figure S1

>pRBD

*AGATCT*AACATCCAAAGACGAAAGGTTGAATGAAACCTTTTTGCCATCCGACATCCACAGGTCCATTCTCACACATAAGTGCCAAACGCAACAGGAGGGGATACACTAGCAGCAGACCGTTGCAAACGCAGGACCTCCACTCCTCTTCTCCTCAACACCCACTTTTGCCATCGAAAAACCAGCCCAGTTATTGGGCTTGATTGGAGCTCGCTCATTCCAATTCCTTCTATTAGGCTACTAACACCATGACTTTATTAGCCTGTCTATCCTGGCCCCCCTGGCGAGGTTCATGTTTGTTTATTTCCGAATGCAACAAGCTCCGCATTACACCCGAACATCACTCCAGATGAGGGCTTTCTGAGTGTGGGGTCAAATAGTTTCATGTTCCCCAAATGGCCCAAAACTGACAGTTTAAACGCTGTCTTGGAACCTAATATGACAAAAGCGTGATCTCATCCAAGATGAACTAAGTTTGGTTCGTTGAAATGCTAACGGCCAGTTGGTCAAAAAGAAACTTCCAAAAGTCGGCATACCGTTTGTCTTGTTTGGTATTGATTGACGAATGCTCAAAAATAATCTCATTAATGCTTAGCGCAGTCTCTCTATCGCTTCTGAACCCCGGTGCACCTGTGCCGAAACGCAAATGGGGAAACACCCGCTTTTTGGATGATTATGCATTGTCTCCACATTGTATGCTTCCAAGATTCTGGTGGGAATACTGCTGATAGCCTAACGTTCATGATCAAAATTTAACTGTTCTAACCCCTACTTGACAGCAATATATAAACAGAAGGAAGCTGCCCTGTCTTAAACCTTTTTTTTTATCATCATTATTAGCTTACTTTCATAATTGCGACTGGTTCCAATTGACAAGCTTTTGATTTTAACGACTTTTAACGACAACTTGAGAAGATCAAAAAACAACTAATTATTCGAAACG**ATG**AGATTTCCTTCAATTTTTACTGCTGTTTTATTCGCAGCATCCTCCGCATTAGCTGCTCCAGTCAACACTACAACAGAAGATGAAACGGCACAAATTCCGGCTGAAGCTGTCATCGGTTACTCAGATTTAGAAGGGGATTTCGATGTTGCTGTTTTGCCATTTTCCAACAGCACAAATAACGGGTTATTGTTTATAAATACTACTATTGCCAGCATTGCTGCTAAAGAAGAAGGGGTATCTCTCGAGAAAAGAGAGGCTG*AAGCT*GCAG*GAATTC*TTGGTGGTTCTTCTGGTGGTTCTAACATTACTAACTTGTGTCCATTTGGTGAAGTTTTTAACGCTACTAGATTTGCTTCTGTTTACGCTTGGAACAGAAAGAGAATTTCTAACTGTGTTGCTGATTACTCTGTTTTGTACAACTCTGCTTCTTTTTCTACTTTTAAGTGTTACGGTGTTTCTCCAACTAAGTTGAACGATTTGTGTTTTACTAACGTTTACGCTGATTCTTTTGTTATTAGAGGTGATGAAGTTAGACAAATTGCTCCAGGTCAAACTGGTAAGATTGCTGATTACAACTACAAGTTGCCAGATGATTTTACTGGTTGTGTTATTGCTTGGAACTCTAACAACTTGGATTCTAAGGTTGGTGGTAACTACAACTACTTGTACAGATTGTTTAGAAAGTCTAACTTGAAGCCATTTGAAAGAGATATTTCTACTGAAATTTACCAAGCTGGTTCTACTCCATGTAACGGTGTTGAAGGTTTTAACTGTTACTTTCCATTGCAATCTTACGGTTTTCAACCAACTAACGGTGTTGGTTACCAACCATACAGAGTTGTTGTTTTGTCTTTTGAATTGTTGCATGCTCCAGCTACTGTTTGTGGTCCAAAGAAGGGTGGTTCTGGTGGTTCTTCTTCTTCTTCTTCTTCTTCTTCTTCT*GGTACCGGCGGCCGCTCTAGA*GAAAACTTGTACTTTCAAGGTGTCGACCATCATCATCATCATCAT**TGA**GTTTGTAGCCTTAGACATGACTGTTCCTCAGTTCAAGTTGGGCACTTACGAGAAGACCGGTCTTGCTAGATTCTAATCAAGAGGATGTCAGAATGCCATTTGCCTGAGAGATGCAGGCTTCATTTTTGATACTTTTTTATTTGTAACCTATATAGTATAGGATTTTTTTTGTCATTTTGTTTCTTCTCGTACGAGCTTGCTCCTGATCAGCCTATCTCGCAGCTGATGAATATCTTGTGGTAGGGGTTTGGGAAAATCATTCGAGTTTGATGTTTTTCTTGGTATTTCCCACTCCTCTTCAGAGTACAGAAGATTAAGTGAGACCTTCGTTTGTGCGGATCCCCCACACACCATAGCTTCAAAATGTTTCTACTCCTTTTTTACTCTTCCAGATTTTCTCGGACTCCGCGCATCGCCGTACCACTTCAAAACACCCAAGCACAGCATACTAAATTTTCCCTCTTTCTTCCTCTAGGGTGTCGTTAATTACCCGTACTAAAGGTTTGGAAAAGAAAAAAGAGACCGCCTCGTTTCTTTTTCTTCGTCGAAAAAGGCAATAAAAATTTTTATCACGTTTCTTTTTCTTGAAATTTTTTTTTTTAGTTTTTTTCTCTTTCAGTGACCTCCATTGATATTTAAGTTAATAAACGGTCTTCAATTTCTCAAGTTTCAGTTTCATTTTTCTTGTTCTATTACAACTTTTTTTACTTCTTGTTCATTAGAAAGAAAGCATAGCAATCTAATCTAAGGGGCGGTGTTGACAATTAATCATCGGCATAGTATATCGGCATAGTATAATACGACAAGGTGAGGAACTAAACCATGGCCAAGTTGACCAGTGCCGTTCCGGTGCTCACCGCGCGCGACGTCGCCGGAGCGGTCGAGTTCTGGACCGACCGGCTCGGGTTCTCCCGGGACTTCGTGGAGGACGACTTCGCCGGTGTGGTCCGGGACGACGTGACCCTGTTCATCAGCGCGGTCCAGGACCAGGTGGTGCCGGACAACACCCTGGCCTGGGTGTGGGTGCGCGGCCTGGACGAGCTGTACGCCGAGTGGTCGGAGGTCGTGTCCACGAACTTCCGGGACGCCTCCGGGCCGGCCATGACCGAGATCGGCGAGCAGCCGTGGGGGCGGGAGTTCGCCCTGCGCGACCCGGCCGGCAACTGCGTGCACTTCGTGGCCGAGGAGCAGGACTGACACGTCCGACGGCGGCCCACGGGTCCCAGGCCTCGGAGATCCGTCCCCCTTTTCCTTTGTCGATATCATGTAATTAGTTATGTCACGCTTACATTCACGCCCTCCCCCCACATCCGCTCTAACCGAAAAGGAAGGAGTTAGACAACCTGAAGTCTAGGTCCCTATTTATTTTTTTATAGTTATGTTAGTATTAAGAACGTTATTTATATTTCAAATTTTTCTTTTTTTTCTGTACAGACGCGTGTACGCATGTAACATTATACTGAAAACCTTGCTTGAGAAGGTTTTGGGACGCTCGAAGGCTTTAATTTGCAAGCTGGAGACCAACATGTGAGCAAAAGGCCAGCAAAAGGCCAGGAACCGTAAAAAGGCCGCGTTGCTGGCGTTTTTCCATAGGCTCCGCCCCCCTGACGAGCATCACAAAAATCGACGCTCAAGTCAGAGGTGGCGAAACCCGACAGGACTATAAAGATACCAGGCGTTTCCCCCTGGAAGCTCCCTCGTGCGCTCTCCTGTTCCGACCCTGCCGCTTACCGGATACCTGTCCGCCTTTCTCCCTTCGGGAAGCGTGGCGCTTTCTCAATGCTCACGCTGTAGGTATCTCAGTTCGGTGTAGGTCGTTCGCTCCAAGCTGGGCTGTGTGCACGAACCCCCCGTTCAGCCCGACCGCTGCGCCTTATCCGGTAACTATCGTCTTGAGTCCAACCCGGTAAGACACGACTTATCGCCACTGGCAGCAGCCACTGGTAACAGGATTAGCAGAGCGAGGTATGTAGGCGGTGCTACAGAGTTCTTGAAGTGGTGGCCTAACTACGGCTACACTAGAAGGACAGTATTTGGTATCTGCGCTCTGCTGAAGCCAGTTACCTTCGGAAAAAGAGTTGGTAGCTCTTGATCCGGCAAACAAACCACCGCTGGTAGCGGTGGTTTTTTTGTTTGCAAGCAGCAGATTACGCGCAGAAAAAAAGGATCTCAAGAAGATCCTTTGATCTTTTCTACGGGGTCTGACGCTCAGTGGAACGAAAACTCACGTTAAGGGATTTTGGTCATGAGATC

Figure S2

>pRBD-SAG1

*AGATCT*AACATCCAAAGACGAAAGGTTGAATGAAACCTTTTTGCCATCCGACATCCACAGGTCCATTCTCACACATAAGTGCCAAACGCAACAGGAGGGGATACACTAGCAGCAGACCGTTGCAAACGCAGGACCTCCACTCCTCTTCTCCTCAACACCCACTTTTGCCATCGAAAAACCAGCCCAGTTATTGGGCTTGATTG*GAGCTC*GCTCATTCCAATTCCTTCTATTAGGCTACTAACACCATGACTTTATTAGCCTGTCTATCCTGGCCCCCCTGGCGAGGTTCATGTTTGTTTATTTCCGAATGCAACAAGCTCCGCATTACACCCGAACATCACTCCAGATGAGGGCTTTCTGAGTGTGGGGTCAAATAGTTTCATGTTCCCCAAATGGCCCAAAACTGACAGTTTAAACGCTGTCTTGGAACCTAATATGACAAAAGCGTGATCTCATCCAAGATGAACTAAGTTTGGTTCGTTGAAATGCTAACGGCCAGTTGGTCAAAAAGAAACTTCCAAAAGTCGGCATACCGTTTGTCTTGTTTGGTATTGATTGACGAATGCTCAAAAATAATCTCATTAATGCTTAGCGCAGTCTCTCTATCGCTTCTGAACCCCGGTGCACCTGTGCCGAAACGCAAATGGGGAAACACCCGCTTTTTGGATGATTATGCATTGTCTCCACATTGTATGCTTCCAAGATTCTGGTGGGAATACTGCTGATAGCCTAACGTTCATGATCAAAATTTAACTGTTCTAACCCCTACTTGACAGCAATATATAAACAGAAGGAAGCTGCCCTGTCTTAAACCTTTTTTTTTATCATCATTATTAGCTTACTTTCATAATTGCGACTGGTTCCAATTGACAAGCTTTTGATTTTAACGACTTTTAACGACAACTTGAGAAGATCAAAAAACAACTAATTATTCGAAACG**ATG**AGATTTCCTTCAATTTTTACTGCTGTTTTATTCGCAGCATCCTCCGCATTAGCTGCTCCAGTCAACACTACAACAGAAGATGAAACGGCACAAATTCCGGCTGAAGCTGTCATCGGTTACTCAGATTTAGAAGGGGATTTCGATGTTGCTGTTTTGCCATTTTCCAACAGCACAAATAACGGGTTATTGTTTATAAATACTACTATTGCCAGCATTGCTGCTAAAGAAGAAGGGGTATCTCTCGAGAAAAGAGAGGCTG*AAGCT*GCAG*GAATTC*TTGGTGGTTCTTCTGGTGGTTCTAACATTACTAACTTGTGTCCATTTGGTGAAGTTTTTAACGCTACTAGATTTGCTTCTGTTTACGCTTGGAACAGAAAGAGAATTTCTAACTGTGTTGCTGATTACTCTGTTTTGTACAACTCTGCTTCTTTTTCTACTTTTAAGTGTTACGGTGTTTCTCCAACTAAGTTGAACGATTTGTGTTTTACTAACGTTTACGCTGATTCTTTTGTTATTAGAGGTGATGAAGTTAGACAAATTGCTCCAGGTCAAACTGGTAAGATTGCTGATTACAACTACAAGTTGCCAGATGATTTTACTGGTTGTGTTATTGCTTGGAACTCTAACAACTTGGATTCTAAGGTTGGTGGTAACTACAACTACTTGTACAGATTGTTTAGAAAGTCTAACTTGAAGCCATTTGAAAGAGATATTTCTACTGAAATTTACCAAGCTGGTTCTACTCCATGTAACGGTGTTGAAGGTTTTAACTGTTACTTTCCATTGCAATCTTACGGTTTTCAACCAACTAACGGTGTTGGTTACCAACCATACAGAGTTGTTGTTTTGTCTTTTGAATTGTTGCATGCTCCAGCTACTGTTTGTGGTCCAAAGAAGGGTGGTTCTGGTGGTTCTTCTTCTTCTTCTTCTTCTTCTTCTTCT*GGTACCGGCGGCCGCTCTAGA*GGTCGGAACCTCGGTACAGCTAGCGCCAAAAGCTCTTTTATCTCAACCACTACTACTGATTTAACAAGTATAAACACTAGTGCGTATTCCACTGGATCCATTTCCACAGTAGAAACAGGCAATCGAACTACATCAGAAGTGATCAGCCATGTGGTGACTACCAGCACAAAACTGTCTCCAACTGCTACTACCAGCCTGACAATTGCACAAACCAGTATCTATTCTACTGACTCAAATATCACAGTAGGAACAGATATTCACACCACATCAGAAGTGATTAGTGATGTGGAAACCATTAGCAGAGAAACAGCTTCGACCGTTGTAGCCGCTCCAACCTCAACAACTGGATGGACAGGCGCTATGAATACTTACATCTCGCAATTTACATCCTCTTCTTTCGCAACAATCAACAGCACACCAATAATCTCTTCATCAGCAGTATTTGAAACCTCAGATGCTTCAATTGTCAATGTGCACACTGAAAATATCACGAATACTGCTGCTGTTCCATCTGAAGAGCCCACTTTTGTAAATGCCACGAGAAACTCCTTAAATTCCTTCTGCAGCAGCAAACAGCCATCCAGTCCCTCATCTTATACGTCTTCCCCACTCGTATCGTCCCTCTCCGTAAGCAAAACATTACTAAGCACCAGTTTTACGCCTTCTGTGCCAACATCTAATACATATATCAAAACGAAAAATACGGGTTACTTTGAGCACACGGCTTTGACAACATCTTCAGTTGGCCTTAATTCTTTTAGTGAAACAGCAGTCTCATCTCAGGGAACGAAAATTGACACCTTTTTAGTGTCATCCTTGATCGCATATCCTTCTTCTGCATCAGGAAGCCAATTGTCCGGTATCCAACAGAATTTCACATCAACTTCTCTCATGATTTCAACCTATGAAGGTAAAGCGTCTATATTTTTCTCAGCTGAGCTCGGTTCGATCATTTTTCTGCTTTTGTCGTACCTGCTATTC**TAA***GTCGAC*GTTTGTAGCCTTAGACATGACTGTTCCTCAGTTCAAGTTGGGCACTTACGAGAAGACCGGTCTTGCTAGATTCTAATCAAGAGGATGTCAGAATGCCATTTGCCTGAGAGATGCAGGCTTCATTTTTGATACTTTTTTATTTGTAACCTATATAGTATAGGATTTTTTTTGTCATTTTGTTTCTTCTCGTACGAGCTTGCTCCTGATCAGCCTATCTCGCAGCTGATGAATATCTTGTGGTAGGGGTTTGGGAAAATCATTCGAGTTTGATGTTTTTCTTGGTATTTCCCACTCCTCTTCAGAGTACAGAAGATTAAGTGAGACCTTCGTTTGTGCGGATCCCCCACACACCATAGCTTCAAAATGTTTCTACTCCTTTTTTACTCTTCCAGATTTTCTCGGACTCCGCGCATCGCCGTACCACTTCAAAACACCCAAGCACAGCATACTAAATTTTCCCTCTTTCTTCCTCTAGGGTGTCGTTAATTACCCGTACTAAAGGTTTGGAAAAGAAAAAAGAGACCGCCTCGTTTCTTTTTCTTCGTCGAAAAAGGCAATAAAAATTTTTATCACGTTTCTTTTTCTTGAAATTTTTTTTTTTAGTTTTTTTCTCTTTCAGTGACCTCCATTGATATTTAAGTTAATAAACGGTCTTCAATTTCTCAAGTTTCAGTTTCATTTTTCTTGTTCTATTACAACTTTTTTTACTTCTTGTTCATTAGAAAGAAAGCATAGCAATCTAATCTAAGGGGCGGTGTTGACAATTAATCATCGGCATAGTATATCGGCATAGTATAATACGACAAGGTGAGGAACTAAACCATGGCCAAGTTGACCAGTGCCGTTCCGGTGCTCACCGCGCGCGACGTCGCCGGAGCGGTCGAGTTCTGGACCGACCGGCTCGGGTTCTCCCGGGACTTCGTGGAGGACGACTTCGCCGGTGTGGTCCGGGACGACGTGACCCTGTTCATCAGCGCGGTCCAGGACCAGGTGGTGCCGGACAACACCCTGGCCTGGGTGTGGGTGCGCGGCCTGGACGAGCTGTACGCCGAGTGGTCGGAGGTCGTGTCCACGAACTTCCGGGACGCCTCCGGGCCGGCCATGACCGAGATCGGCGAGCAGCCGTGGGGGCGGGAGTTCGCCCTGCGCGACCCGGCCGGCAACTGCGTGCACTTCGTGGCCGAGGAGCAGGACTGACACGTCCGACGGCGGCCCACGGGTCCCAGGCCTCGGAGATCCGTCCCCCTTTTCCTTTGTCGATATCATGTAATTAGTTATGTCACGCTTACATTCACGCCCTCCCCCCACATCCGCTCTAACCGAAAAGGAAGGAGTTAGACAACCTGAAGTCTAGGTCCCTATTTATTTTTTTATAGTTATGTTAGTATTAAGAACGTTATTTATATTTCAAATTTTTCTTTTTTTTCTGTACAGACGCGTGTACGCATGTAACATTATACTGAAAACCTTGCTTGAGAAGGTTTTGGGACGCTCGAAGGCTTTAATTTGCAAGCTGGAGACCAACATGTGAGCAAAAGGCCAGCAAAAGGCCAGGAACCGTAAAAAGGCCGCGTTGCTGGCGTTTTTCCATAGGCTCCGCCCCCCTGACGAGCATCACAAAAATCGACGCTCAAGTCAGAGGTGGCGAAACCCGACAGGACTATAAAGATACCAGGCGTTTCCCCCTGGAAGCTCCCTCGTGCGCTCTCCTGTTCCGACCCTGCCGCTTACCGGATACCTGTCCGCCTTTCTCCCTTCGGGAAGCGTGGCGCTTTCTCAATGCTCACGCTGTAGGTATCTCAGTTCGGTGTAGGTCGTTCGCTCCAAGCTGGGCTGTGTGCACGAACCCCCCGTTCAGCCCGACCGCTGCGCCTTATCCGGTAACTATCGTCTTGAGTCCAACCCGGTAAGACACGACTTATCGCCACTGGCAGCAGCCACTGGTAACAGGATTAGCAGAGCGAGGTATGTAGGCGGTGCTACAGAGTTCTTGAAGTGGTGGCCTAACTACGGCTACACTAGAAGGACAGTATTTGGTATCTGCGCTCTGCTGAAGCCAGTTACCTTCGGAAAAAGAGTTGGTAGCTCTTGATCCGGCAAACAAACCACCGCTGGTAGCGGTGGTTTTTTTGTTTGCAAGCAGCAGATTACGCGCAGAAAAAAAGGATCTCAAGAAGATCCTTTGATCTTTTCTACGGGGTCTGACGCTCAGTGGAACGAAAACTCACGTTAAGGGATTTTGGTCATGAGATC
